# Genome Sequence of *Bradyrhizobium stylosanthis* Strain BR 446^T^, a Nitrogen-Fixing Symbiont of the Legume Pasture *Stylosanthes guianensis*

**DOI:** 10.1128/genomeA.00631-16

**Published:** 2016-06-30

**Authors:** Jakeline Renata Marçon Delamuta, Renan Augusto Ribeiro, Douglas Fabiano Gomes, Renata Carolini Souza, Ligia Maria Oliveira Chueire, Mariangela Hungria

**Affiliations:** a Embrapa Soja, Soil Biotechnology, Londrina, Paraná, Brazil; b CAPES, SBN, Edifício Capes, Brasília, Federal District, Brazil; c CNPq, SHIS QI 1 Conjunto B, Lago Sul, Brasília, Federal District, Brazil

## Abstract

*Bradyrhizobium stylosanthis* BR 446^T^ is a nitrogen-fixing symbiont of the tropical legume pasture *Stylosanthes guianensis*. Its draft genome contains 8,801,717 bp and 8,239 coding sequences (CDSs). Several putative genes that might confer high competitiveness and saprophytic capacity under the stressful conditions of tropical soils were identified in the genome.

## GENOME ANNOUNCEMENT

Several legumes stand out as economically important crops, however, legume forages are receiving increasing attention, especially in tropical regions, because of their potential in improving soil fertility. Benefits of using legumes rely mainly on their capacity of fixing atmospheric nitrogen with bacteria collectively known as rhizobia (1, 2).

*Bradyrhizobium* encompasses several bacteria highly effective in fixing nitrogen, but although the genus represents the most important group of rhizobia in the tropics, our knowledge about their diversity is still poor (1, 3–5). Our group has reported several studies on the diversity of *Bradyrhizobium* strains in Brazil (3, 4, 6), and some of them derived in the description of new species (7–9). Recently, we have described the new species *Bradyrhizobium stylosanthis*, including strains isolated from *Stylosanthes* spp., an important perennial tropical forage legume (10). Now we report the draft genome of the type strain of this species, BR 446^T^ (=CNPSo 2823^T^ =HAMBI 3668^T^ =H-8^T^).

To access the bacterial genome sequence, total DNA was extracted using the DNeasy blood and tissue kit (Qiagen) and processed on the MiSeq plataform (Illumina) at Embrapa Soja, Londrina, Brazil. Shotgun sequencing generated 613,771 paired-end reads (2 × 300 bp), corresponding to approximately a 20.45-fold coverage. The FASTQ files were *de novo* assembled by A5-miseq pipeline, which performs read trimming, contig assembly, misassembly correction, and final scaffolding (11). The genome analyses revealed that strain BR 446^T^ has one circular chromosome and a G+C content of 63.88 mol%. Sequences were submitted to RAST (12) and the genome is estimated to be 8,801,717 bp, assembled in 22 contigs, with 8,239 predicted coding sequences (CDSs).

The analysis using the SEED system (12) allowed the classification of 40% of the CDSs in 512 subsystems. In the symbiosis, nodulation is a key process and the genes that might confer higher or lower competitiveness to one strain are still not well understood. Interestingly, in BR 446^T^, 123 CDSs fit into the category of virulence, disease, and defense, including 13 beta-lactamase, bacteriocins, and a whole *Mycobacterium* virulence operon. There were also 27 CDSs related to iron acquisition and metabolism and 218 CDSs related to motility and chemotaxis, including several putative genes that might help with higher competitiveness. In relation to the nodulation and nitrogen fixation genes, they are in a putative symbiotic island, but we found only one copy of the regulatory *nodD*. In addition, *nif* operons were more conserved compared to other *Bradyrhizobium* strains than the *nod* operons. A variety of CDSs related to stress response (205) might be implicated in the high saprophytic capacity of the strain under the stressful conditions of tropical soils. The information obtained with the genome of *B. stylosanthis* contributes to the knowledge of the diversity of tropical rhizobia, as well as to studies on taxonomy and phylogeny of rhizobia.

### Nucleotide sequence accession numbers.

This whole-genome shotgun project has been deposited at DDBJ/EMBL/GenBank under the accession number LVEM00000000, SUBID SUB1384295, BioProject PRJNA315153, BioSample SAMN04549886. The version described in this paper is LVEM01000000.
